# Unbiased profiling of volatile organic compounds in the headspace of *Allium* plants using an in-tube extraction device

**DOI:** 10.1186/s13104-016-1942-5

**Published:** 2016-02-29

**Authors:** Miyako Kusano, Makoto Kobayashi, Yumiko Iizuka, Atsushi Fukushima, Kazuki Saito

**Affiliations:** Graduate School of Life and Environmental Sciences, University of Tsukuba, Tennodai, Tsukuba, Ibaraki Japan; RIKEN Center for Sustainable Resource Science, Yokohama, Kanagawa Japan; Department of Genome System Science, Graduate School of Nanobioscience, Yokohama City University, Yokohama, Kanagawa Japan; Graduate School of Pharmaceutical Sciences, Chiba University, Chiba, Chiba Japan

**Keywords:** Volatile organic compounds, GC-TOF–MS, Metabolomics, Headspace, ITEX, *Allium*

## Abstract

**Background:**

Plants produce and emit important volatile organic compounds (VOCs), which have an essential role in biotic and abiotic stress responses and in plant–plant and plant–insect interactions. In order to study the bouquets from plants qualitatively and quantitatively, a comprehensive, analytical method yielding reproducible results is required.

**Results:**

We applied in-tube extraction (ITEX) and solid-phase microextraction (SPME) for studying the emissions of *Allium* plants. The collected HS samples were analyzed by gas chromatography–time-of-flight–mass spectrometry (GC-TOF–MS), and the results were subjected to multivariate analysis. In case of ITEX-method *Allium* cultivars released more than 300 VOCs, out of which we provisionally identified 50 volatiles. We also used the VOC profiles of *Allium* samples to discriminate among groups of *A. fistulosum*, *A. chinense* (rakkyo), and *A. tuberosum* (Oriental garlic). As we found 12 metabolite peaks including dipropyl disulphide with significant changes in *A. chinense* and *A. tuberosum* when compared to the control cultivar, these metabolite peaks can be used for chemotaxonomic classification of *A.* c*hinense*, *tuberosum*, and *A. fistulosum*.

**Conclusions:**

Compared to SPME-method our ITEX-based VOC profiling technique contributes to automatic and reproducible analyses. Hence, it can be applied to high-throughput analyses such as metabolite profiling.

**Electronic supplementary material:**

The online version of this article (doi:10.1186/s13104-016-1942-5) contains supplementary material, which is available to authorized users.

## Background

Plants produce various kinds of volatile organic compounds (VOCs) that are a part of the metabolome. By today the total number of identified VOCs is about 1700, and they account for 1 % of secondary metabolites [1, 2]. The major chemical classes of VOCs emitted from plants are terpenoids, phenylpropanoids/benzenoids, and derivatives of fatty acids and amino acids [3]. The genus *Allium*, is comprised of onions, leeks, and garlic, the total number of species is to 600–750 [4]. *Allium* plants can produce sulfur-containing VOCs through enzymatic reaction of sulfur-storage compounds [4]. For example, primary “aroma” compounds are thiosulfinates including allicin that are produced from aliphatic cysteine sulfoxides as “aroma” precursors in Genus *Allium*. Dithiins, ajoenes, and sulfides are known to as secondary “aroma” compounds [5].

We chose *Allium fistulosum* (Japanese bunching onions), *A. chinense* (rakkyo), and *A. tuberosum* (Oriental garlic), because these plants have been cultivated in Japan since the 8th century and are favorites of the Japanese. *Allium* plants emit VOCs that result in strong odors. The odoriferous compounds whose moiety contains sulfur in their moieties function not only as a defense against pathogens [6] and insects [7], but they also attract special herbivores and insect-eating insects such as moths [8, 9] and bees [10]. The chemical composition of such metabolites is diverse [11]. Since sulfur-containing VOCs produced by *Allium* plants exhibit anticancer- [12, 13], antithrombotic- [14], and antibacterial activity [15, 16], they are thought to be beneficial to human health.

There are several methods to collect VOCs in various matrices. A traditional way is steam distillation by which oils produced by plants can be collected. Meanwhile headspace (HS) sampling is a non-destructive solvent-free method for collecting VOCs emitted from plants [17, 18] including vegetables [19], humans [20], and microbes [21]. Moreover, the investigation of HS composition is much more meaningful than volatile analysis of samples collected by distillation or extraction methods. In case of high concentration capacity HS (HCC–HS) sampling methods [17, 22] such as solid-phase microextraction (SPME) [23–25], in-tube extraction (ITEX) [26–28], and stir bar sportive extraction (SBSE) [29, 30], VOCs can be easily concentrated. However, there is still a need for developing a comprehensive, reproducible, and high-throughput analysis for detection and quantification of VOCs in biological samples of various cultivars. Less than approximately 20 samples can be analyzed as one batch with one SPME fiber due to capacity of sorbent materials of SPME. Trapping of VOCs depends on SPME fibers’ properties [31]. To date, many types of sorbent materials are commercially available for ITEX-based method. Choosing the appropriate sorbent material of ITEX is important to trap non-polar and/or polar VOCs. Compared to SPME-method sampling according to ITEX procedure is fully-automated at the four steps, i.e., sample conditioning, analyte extraction/sorption, desorption/injection, and trap conditioning. Plus more samples can be analyzed by using ITEX- than SPME-method [32]. As high-throughput analysis is required for VOC profiling, we applied ITEX method in this study.

After HCC-HS sampling, VOCs are directly analyzed by gas chromatography (GC)-based techniques, because target analytes are easily released by heating sorbent materials. Of these methods, GC combined with electron ionization–time-of-flight–mass spectrometry (EI-TOF–MS) may help to identify and estimate the structure of VOCs, because EI-TOF–MS yields comprehensive information on molecular fragments in terms of mass-to-charge ratios [33], and because of well-documented libraries such as NIST/EPA/NIH mass spectral library (NIST-L) [34], Adams library (Ad-L) [35], the terpenoids library (Te-L; http://www.massfinder.com/wiki/Terpenoids_Library), and VocBinbase (Vo) [29], which contain mass spectral and retention index (RI) information of compounds that can be analyzed by GC–MS. Furthermore, several alignment tools such as AMDIS [36], ChromA [37], H-MCR [38], metalign [39], Tagfinder [40], and XCMS [41] have been developed and are freely available for GC–MS data interpretation.

The goal of the study was to develop a comprehensive, reproducible, and high-throughput profiling method for VOC collection from many samples by using fully-automated ITEX procedure and to then provisionally identify the detected VOCs in the HS of plants using the summarized mass spectral libraries. By applying our pipeline, we performed comprehensive HS-VOC profiling of the sheaths and basal plates of 12 *Allium* cultivars with ITEX-method in this study.

## Results and discussion

### Optimization of HCC-HS sampling and comparison of HS-VOC profiles in the HS of *Allium fistulosum* using ITEX and SPME techniques

To achieve the best performance for HCC-HS sampling in HS-GC vials, a method is needed that suits the goal of comprehensive, reproducible analyses. To this end, we modified the method of Tikunov et al. [23] and Kusano et al. [31]. *Allium* plants produce sulfides such as dipropyl disulfide as the main VOC component [7, 42]. We used ITEX and SPME to conduct HCC-HS collection from the *Allium* plants and evaluated statistically. The choice of the internal standards (ISs) is also critical for non-targeted metabolite profiling [43, 44]. Several ISs with different physicochemical properties (i.e., RI and chemical structure) are required for a comprehensive VOC analysis to evaluate whether the analytes participate in cross-contribution [44] and whether the RI of each IS peak is reproducible. Therefore we carefully examined dissolving agents for the ISs based on the value of the partition coefficient and the solubility of each IS [45] and selected methanol as the solvent.

**Fig. 1:**
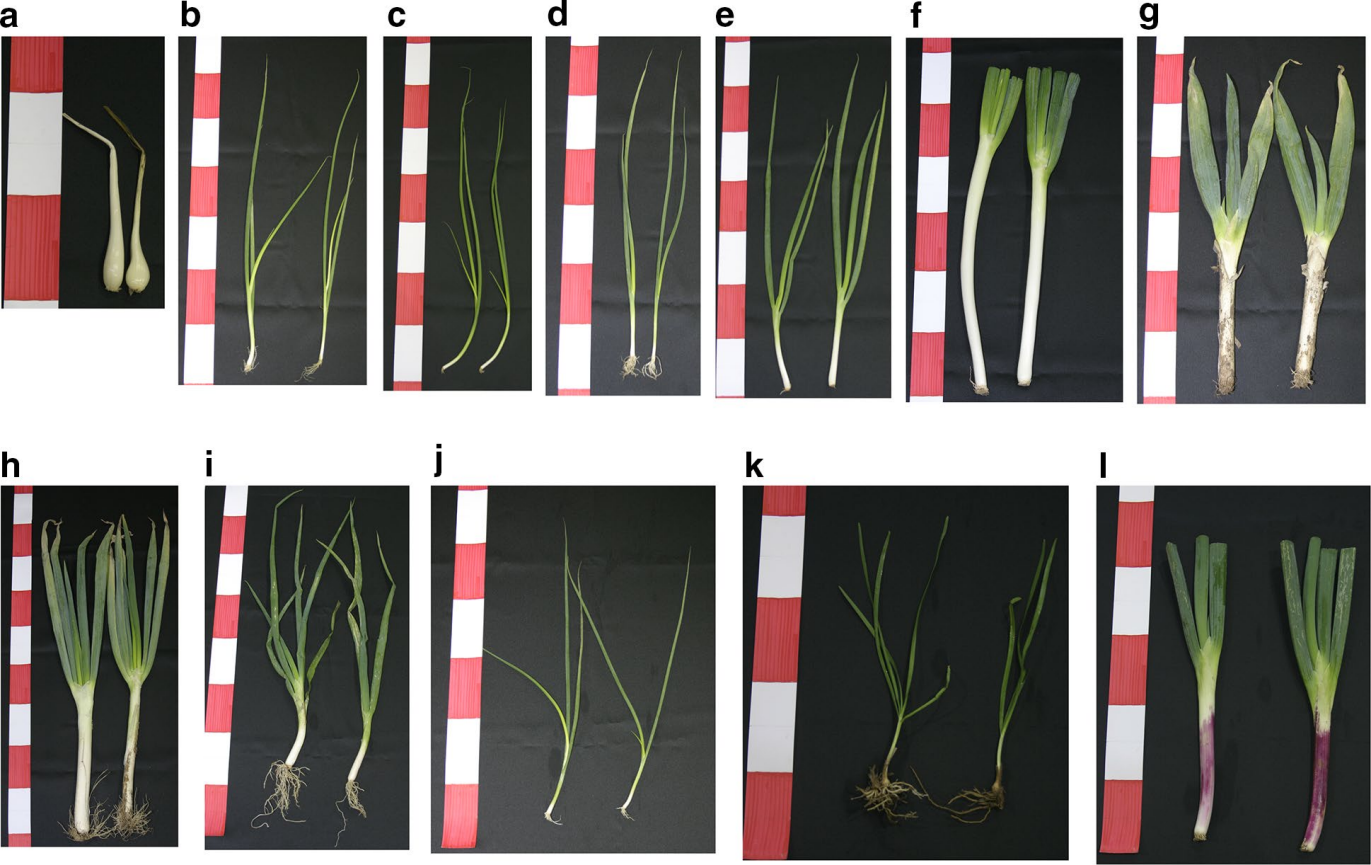
Visual phenotypes of the *Allium* samples used in this study. **a** *A. chinense* (rakkyo, class08 in Table 1 and Fig. 4). **b** *A. fistulosum* (spring onion, class01), **c** *A. fistulosum* (green spring onion, class 02), **d** *A. fistulosum* (scallion, class03), **e** *A. fistulosum* (green spring onion, class04), **f** *A. fistulosum* (white spring onion, class05), **g** *A. fistulosum* (leek, class06), **h** *A. fistulosum* (leek, class07), **i** *A. fistulosum* (scallion, class09), **j** *A. fistulosum* (spring onion, class11), **k** *A. tuberosum* (Oriental garlic, class10), **l** *A. fistulosum* (red spring onion, class12). The *red* and *white* areas of the *scale bar* are 5 cm

We conducted HCC-HS sampling using ITEX and SPME to compare their performance for peak detection and to assess their comprehensiveness and the reproducibility of the results obtained with each technique. First we estimated the lower limit of quantification (LLOQ) of dipropyl disulfide, the major disulfide in *A. fistulosum* [7] and *A. cepa* [46], using ITEX- and SPME-GC-TOF–MS (see Additional file 2). The LLOQ of the peak detected by ITEX-GC-TOF–MS analysis was 250 pmol; it was 25 pmol by SPME-GC-TOF–MS analysis (data not shown). Then, using both methods, we analyzed the sheath and the basal part of *A. fistulosum* (brand name, Mikata spring onion; class01 in Table 1, Fig. 1, Additional file 1). The total ion chromatogram (TIC) of each analyte showed that peak detection was more sensitive with SPME device (Additional file 1). The score scatter plot of samples analyzed with the ITEX device and the SPME fiber showed clear separation of the first principal component (Additional file 1). It may be due to the use of different resins (TGR/CSIII for ITEX and PDMS/DVB for SPME).

**Table 1:** *Allium* species used in this study

Class in PCA	Binomial name	Species name	Bland name	Harvested field in Japan
08	*Allium chinense*	Rakkyo	Young rakkyo	Namegata, Ibaraki
01	*Allium fistulosum*	Spring onion	Mikata spring onion	Hamamatsu, Shizuoka
02	*Allium fistulosum*	Green spring onion	Aoi-chan green spring onion	Akitakata, Hiroshima
03	*Allium fistulosum*	Scallion	Hakata scallion	Hakata, Fukuoka
04	*Allium fistulosum*	Green spring onion	Green spring onion from Nagareyama	Nagareyama, Chiba
05	*Allium fistulosum*	White spring onion	White spring onion from Nagano	Nagano
06	*Allium fistulosum*	Leek	Shimonita leek	Gunma
07	*Allium fistulosum*	Leek	Shirakami leek	Noshiro, Akita
09	*Allium fistulosum*	Scallion	Kujo scallion	Nagahama, Shiga
11	*Allium fistulosum*	Spring onion	Goudo spring onion	Anpachi, Gifu
10	*Allium tuberosum*	Oriental garlic	Oriental garlic	Nagahama, Shiga
12	*Allium fistulosum*	Red spring onion	Red spring onion	Tsuruoka, Yamagata

All samples were harvested in October 2012

We used the same class names in the PCA score scatter plot (see Fig. 4)

An advantage of the use of ITEX lies in its use of a stainless-steel needle and a special purge-, trap-, and trap-cleaning system [27, 47, 48]. This makes it possible to run more samples while maintaining the high reproducibility of data. On the other hand, the SPME method is appropriate for semi-targeted analysis because it features a wide variety of fibers rather than the ITEX sorbent materials. Although the SPME showed more sensitivity than ITEX to detect VOCs, less than approximately 20 samples can be analyzed as one batch due to capacity of sorbent materials of SPME. Thus, we applied the ITEX method for non-targeted HS-VOC profiling of the *Allium* samples.

### Comparison of the libraries for the tentative identification of HS-VOCs

**Fig. 2:**
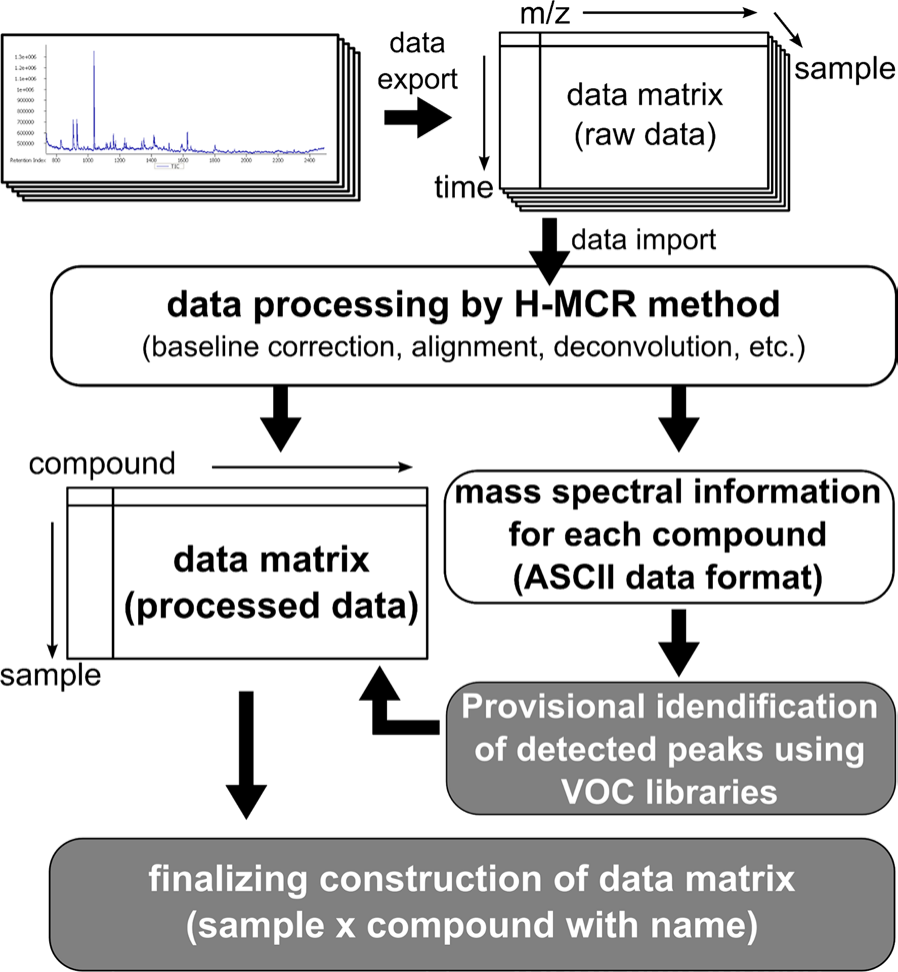
Schema of the workflow for data processing and peak annotation to obtain the data matrix. Non-processed data for GC-TOF–MS analysis of each sample were exported as NetCDF files. These files were imported in MATLAB for baseline correction, peak alignment, and deconvolution by the H-MCR method. Libraries were prepared for the provisional identification of the extracted mass spectra of the VOC peaks (*gray box*). After merging the information into a data matrix, we obtained a data matrix comprised of the compound name, sample name, and the sum of the peak area of each extracted mass

We estimated how many volatile peaks in the mass spectra overlapped in NIST-L, Ad-L, Te-L, and in Vo before provisional identification of the detected peaks. Non-processed MS data from the HS-ITEX-GC-TOF–MS analysis can be exported and then processed using our method for metabolite profiling (Fig. 2). However, the putative identification of the detected VOCs is limited because few libraries show the EI mass spectra and RI and because it is very difficult to obtain authentic standards for VOCs. Despite this limitation, we estimated how many mass spectra overlapped among Vo and the three commercially-available libraries for volatiles (Ad-L, Te-L, NIST05). The estimation procedure is clarified in details in the Materials and Methods section. Instead of complete matching of the compounds using CAS numbers and/or compound names, we used the similarity of each mass spectrum and the RI difference of the corresponding peak in the query library (Ad-L, Te-L, Vo) and the NIST05 reference library (Tables 2 and 3). Approximately 35 % of the mass spectra in Ad-L (3rd edition, 555/1607; 4th edition, 765/2205) exhibited high similarity against NIST05. On the other hand, only four compounds (*β*-maaliene, methyl tridecanoate, methyl undecanoate, and methyleugenol) showed a similarity value greater than 900 in Te-L; the SD of the RI differences of the four compounds was 5.6 (Table 3). Using their chemical structures in NIST05 we compared these compounds and found that they were identical in Ad-L and NIST05. The mass spectra in Te-L tend to be unique. Consequently, the difference shown in Table 3 may increase the number of compounds that can be annotated.

**Table 2:** Libraries used for the provisional identification of VOCs

Library name	RI	Phase composition of the GC column(s)
Adams library (3rd ed.)	Available	5 % diphenyl, 95 % dimethyl polysiloxane
Adams library (4th ed.)	Available	5 % diphenyl, 95 % dimethyl polysiloxane
Terpenoids library	Available	100 % dimethyl polysiloxane
VocBinBase	Available	5 % diphenyl, 95 % dimethyl polysiloxane
NIST05	Available	Various types (polar and non-polar)

**Table 3:** Estimation of the number of similar compounds in the Adams (Ad-L) and the Terpenoids library (Te-L), and in VocBinBase (Vo) against NIST05

Library name	Number of EI spectra	Number of identified compounds	≥850^a^	SD of RI diff (≥850)	≥900^a^	SD of RI diff (≥900)
Ad-L (3rd ed.)	1607	1607	794	8.36	555	8.29
Ad-L (4th ed.)	2205	2205	1077	8.73	765	8.69
Te-L	1982	1982	91	8.48	4	5.56
Vo^b^	1632	212	258	8.67	143	8.09
NIST05	190,825	163,198	–	–	–	–

The RI difference (diff) was calculated by subtracting the RI of a compound peak in the query library from that in the reference library (NIST05). The values were transformed into absolute values

*SD* standard deviation; *RI diff* absolute RI difference

^a^The value represents similarity defined as described in “Methods”

^b^VocBinBase contains 1420 unidentified EI spectra

### HS-VOC profiling of the 12 *Allium* plants using the ITEX technique

**Fig. 3:**
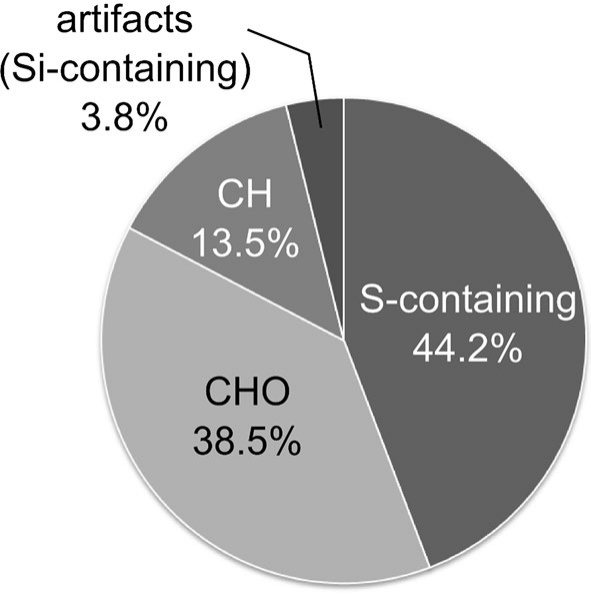
Proportion of sulfur-containing peaks in the 52 annotated peaks, including two artifacts (Si-containing peaks), in the HS of *Allium* plants. The proportion was calculated by counting the number of annotated compounds that consisted of CHOS, CHO, CH, or CHOSi

We conducted VOC profiling in the HS of 10 *A. fistulosum*-, one *A. chinense*-, and one *A. tuberosum* cultivars with the ITEX technique. The visual phenotypes of each *Allium* plant are presented in Fig. 1. We focused on the sheaths and basal plates to analyze the VOCs. The entire aerial parts of the other *Allium* cultivars used in this study are eaten in Japan. We obtained VOC profile data on 35 samples [three biological replicates except for *A. fistulosum* (class05, *n* = 2)] and 354 extracted mass spectral peaks as a data matrix. The detected peaks were identified or provisionally identified using our fully-automated annotation pipeline. Of these, 52 peaks, including two artifacts (Si-containing peaks derived from column breeding) were tentatively identified by comparing their mass spectra and the RI corresponding to those in the four libraries (Table 2), or identified using authentic standards (Additional file 3). The molecular formula of each annotated peak was investigated and the proportion of sulfur-containing peaks in the annotated peaks was calculated (Fig. 3). Approximately half of the annotated peaks contained sulfur atom(s) in their moieties. According to Pino et al. [49], sulfur-containing compounds account for approximately 90 % of the total volatile content in diethyl ether extracts of *A*. *chinense* and *A. tuberosum*. Our findings suggest that ITEX-based VOC profiling could detect not only sulfur-containing peaks but also other types of VOCs.

**Fig. 4:**
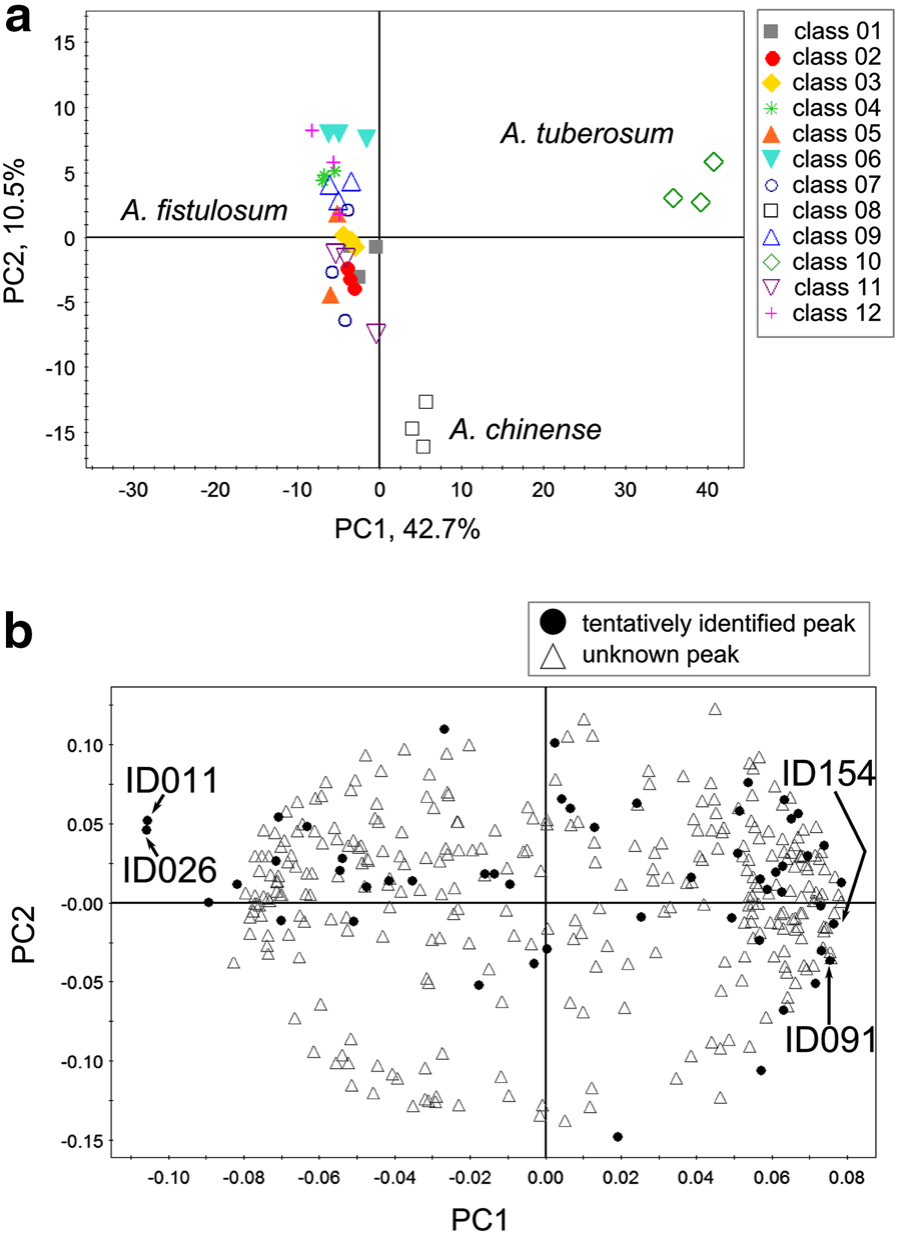
Score (**a**) and loading (**b**) *plots* of PCA of the VOC profiles of the *Allium* samples. Principal components one and two (PC1, PC2) represent the first two principal components that account for a total of 53.2 % of the variance. Each *plot* represents an independent plant. In the loading plot, *black dots* and *white triangles* represent tentatively identified- and unknown peaks, respectively. All compound names and IDs are listed in the Additional file 3. *ID011* 2-butenal, 2-ethyl; *ID026* 3,4-dimethylthiophene; *ID091* diallyl disulphide; *ID154* 2,5-thiophenedicarboxaldehyde

We conducted principal component analysis (PCA) to visualize the similarities/differences in the VOC composition of each *Allium* cultivar (Fig. 4). The score scatter plot of the VOC profile data showed subspecies-dependent separations among *A. chinense*, *A. fistulosum*, and *A. tuberosum* (Fig. 4a). Next, we investigated the distribution of tentatively identified peaks in the profiles of the *Allium* cultivars. The PCA loading plot showed that, some peaks tended to be abundant in *A. fistulosum* cultivars [e.g. 3,4-dimethylthiophene (ID026)], while the levels of the two sulfur-containing compounds [2,5-thiophenedicarboxaldehyde (ID154) and diallyl disulphide (ID091)] were more abundant in *A. tuberosum* than in *A. fistulosum* cultivars (Fig. 4b).

### Discriminative VOCs among the *Allium* cultivars

We compared the VOC profiles of each *Allium* cultivar to determine whether the VOC composition in the HS can be used in their differentiation. VOC changes in the HS of *Allium* samples were recorded by subtracting the average of the normalized responses of the annotated peaks (log_2_-transformed value) in each *Allium* cultivar from those of the control, Mikata spring onion (class01, Fig. 1b). The extent of the VOC changes tended to be similar to that shown by PCA (Fig. 4, Additional file 3). For example, the visual phenotype of the control cultivar Mikata spring onion (class01), and of Aoi-chan green spring onion (class02) was very similar (Fig. 1b, c). There was no significant difference in the level of the annotated VOCs between these cultivars (Additional file 3). In the VOC profiles of other cultivars of *A. fistulosum*, there were a few differences in the VOC levels when compared to the control (data not shown). Thus, we focused on the subspecies-dependent differences.

**Fig. 5:**
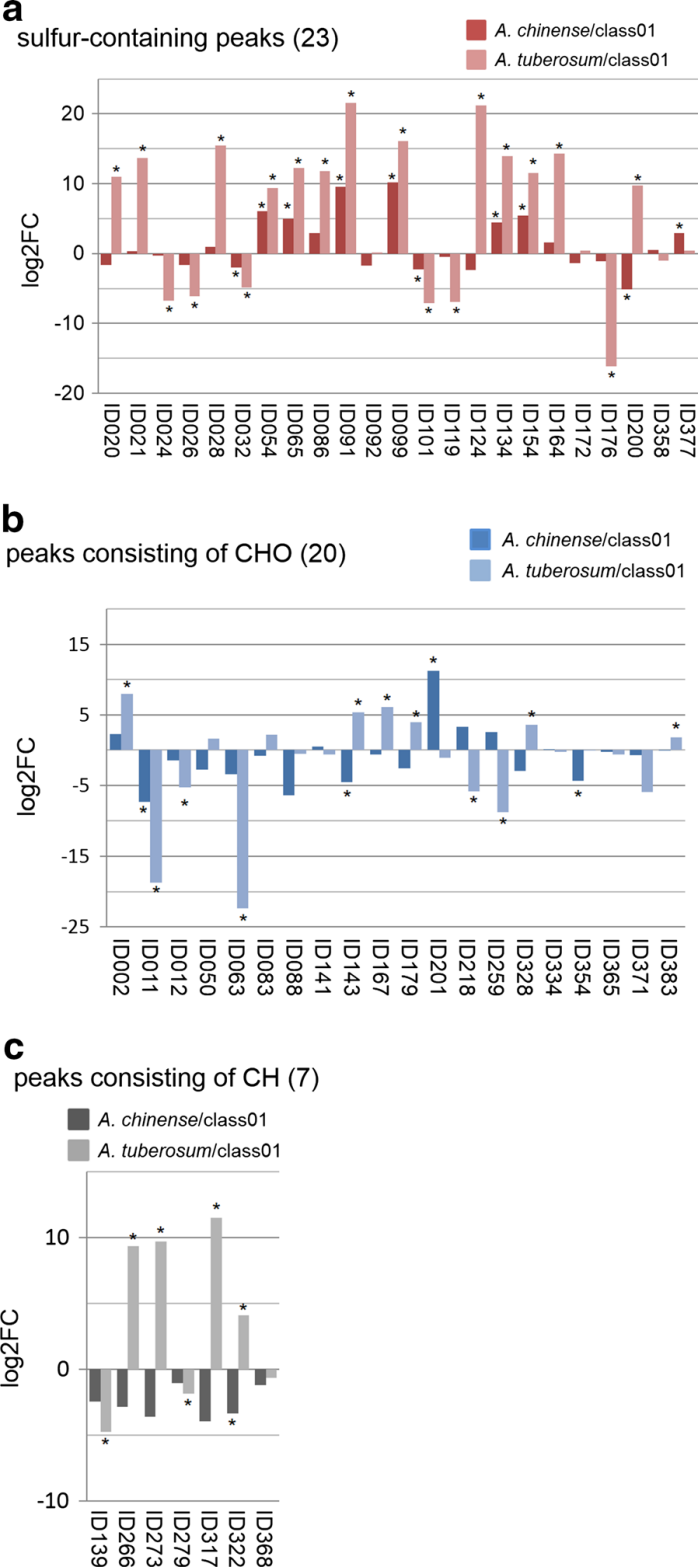
The log_2_-fold changes in the VOCs of 50 annotated peaks in the VOC profiles of sulfur-containing peaks (**a**), peaks consisting of CHO (**b**), and peaks consisting of CH (**c**). The log_2_-fold changes (log2FC) in the normalized response of peaks of each cultivar (*A*. *chinense* or *A*. *tuberosum*) against that of the control cultivar class01 (*A. fistulosum*) are shown in the Additional file 3. We analyzed three biological replicates of each *Allium* plant except the white spring onion (class05, *n* = 2) cultivar. *Asterisks* on the top of the *bars* present that the level of VOCs in *A. chinense* and/or *A. tuberosum* was significantly changed using the LIMMA package (see the “Methods” section) when compared to the control cultivar (class01 in Table 1). The significance level was set at FDR <0.05 (*asterisk* *). Sulfur-containing peaks were: *ID020* 1-propene, 3,3’-thiobis, *ID021* methanesulfonic acid, methyl ester, *ID024* thiophene, 2,5-dimethyl, *ID026* 3,4-dimethylthiophene, *ID028* thiophene, 2-ethenyl, *ID032* thiophene, 2,4-dimethyl, *ID054* dimethyl trisulfide, *ID065* 3-thiophenecarboxaldehyde, *ID086* *S*-methyl methanethiosulphonate, *ID091* diallyl disulphide, *ID092* 5-methyl-2-thiophenecarboxaldehyde, *ID099* 1,2,4-trithiolane, *ID101* dipropyl disulfide, *ID119* prop-1-enyl dithiopropanonate, *ID124* thiophene, 2-propyl, *ID134* 3-vinyl-1,2-dithiacyclohex-5-ene, *ID154* 2,5-thiophenedicarboxaldehyde, *ID164* trisulfide, di-2-propenyl, *ID172* trisulfide, dipropyl, *ID176* 1,2,4-trithiolane, 3,5-diethyl, *ID200* thieno[2,3-b]thiophene, 2-methyl, *ID358* cyclic octaatomic sulfur, *ID377* disulfide, methyl 1-propenyl, Peaks consisting of CHO: *ID002* hexanal, *ID011* 2-butenal, 2-ethyl, *ID012* 2-pentenal, 2-methyl, *ID050* heptenal, (2*E*), *ID063* 2-furanone, 2,5-dihydro-3,5-dimethyl; *ID083* octen-1-al, (2*E*); *ID088* 2-octen-1-ol, (*E*); *ID141* nonanoic acid; *ID143* decenal, (2*E*); *ID167* decadienal, (2*E*,4*E*); *ID179* 2-dodecenal, (*E*); *ID201* 3(2*H*)-furanone, 2-hexyl-5-methyl; *ID218* 2-tridecanone; *ID259* 3(2*H*)-furanone, 5-methyl-2-octyl; *ID328* hexadecanoic acid methyl ester, *n; ID334* 1,2-benzenedicarboxylic acid, butyl octyl ester; *ID354* 9,12-octadecadienoic acid, methyl ester, (*E*,*E*); *ID365* [1,1′,3′,1′-terphenyl]-2′-ol; *ID371* 2,5-cyclohexadiene-1,4-dione, 2,5-diphenyl; *ID383* benzaldehyde; Peaks consisting of CH: *ID139* benzene, 1,3-bis(1,1-dimethylethyl); *ID266* 8-heptadecene; *ID273* heptadecane, *n; ID279* 1,1′-biphenyl, 2,2′,5,5′-tetramethyl; *ID317* 5-octadecene, (*E*); *ID322* nonadecane; *ID368* tricosane

We compared changes in the level of the 50 annotated VOC peaks in the profiles of *A. chinense* and *A. tuberosum* against the control (class01) (false discovery rate, FDR < 0.05). Of these, The 15 compound peaks showed significant changes in the profiles of *A. chinense*, while the level of 36 peaks was changed in *A. tuberosum* (Fig. 5).

Among 23 sulfur-containing peaks, 10 peaks showed significant changes in *A. chinense*, while 19 peaks were significantly changed in *A. tuberosum* (Fig. 5a). Of these, there were nine discriminant peaks in both subspecies. Thiosulfinates are the initial compounds in the HS of *Allium* species when their tissues are chopped or homogenated [50]; they decompose immediately and then sulfides are emitted as major aroma compounds [11, 42]. HS-ITEX VOC profiling detected a monosulfide (ID020), two disulfides (ID091 and ID101), and two trisulfides (ID054 and ID164). Their level was higher in *A. tuberosum* than in the control except for dipropyl disulfide (ID101) that is the major disulfide in *Allium* plants. The level of this compound was significantly lower in *A. chinense* and *A. tuberosum* than in the control (Fig. 5a). Interestingly, the level of this compound differed among the cultivars of *A. fistulosum* (Additional file 3). It suggests that this compound can be used for the chemotaxonomic classification of *A.fistulosum* cultivars. For bunching onions like *A.fistulosum*, DNA markers such as simple sequence repeats (SSRs), amplified fragment length- and single-nucleotide polymorphisms (AFLPs, SNPs) are available (http://www.vegmarks.nivot.affrc.go.jp/VegMarks/jsp/index.jsp). However, its high cost hampers the data collection of many cultivars. As a first step, our VOC profiling is useful for choosing representative cultivars in *Allium* plants for further analyses.

We detected nine peaks that are considered to be thiophene compounds [thiophene, 2,5-dimethyl, 3,4-dimethylthiophene, thiophene, 2,4-dimethyl, thiophene, 2,4-dimethyl, 3-thiophenecarboxaldehyde, thiophene, 2-propyl, 2,5-thiophenedicarboxaldehyde, 1,2,4-trithiolane, 3,5-diethyl, and thieno(2,3-b)thiophene, 2-methyl] in the *Allium* samples. Of these, two dimethylthiophenes (thiophene, 2,5-dimethyl, and 3,4-dimethylthiophene) were found to be thermal decomposition products from dialkyl disulfides in the distilled oils of *Allium* species [42, 51]. In the HS of *Allium* cultivars next to sulfur-containing volatiles there were VOCs, which consisted of CHO atoms. Out of those VOCs five compounds (hexanal, 2-pentenal, 2-methyl, heptenal, (2*E*), 2-tridecanone, and 3(2*H*)-furanone, 5-methyl-2-octyl) are previously found in distilled oils of *A. fistulosum* cultivars [52]. The level of four compound peaks such as hexanal, 2-butenal, 2-ethyl-, 2-tridecanone, and 3(2*H*)-furanone, 5-methyl-2-octyl was significantly different in the profiles of *A. chinense,* while 11 VOCs with asterisk (*) on the top of the bars were significantly changed in the profiles of *A. tuberosum* (Fig. 5b).

Annotated or identified compounds whose moieties included only CH atoms were categorized as alkanes or alkenes (Fig. 5c), out of which odd-numbered alkanes (heptadecane and nonadecane) are previously found in the methanol extract of garlic (*A. sativum*) [53] and in the HS of flowers of heliotrope and mandarin [54, 55]. The function(s) and biosynthetic pathway(s) of such compounds remain largely limited, except for dipropyl trisulphide in *A. fistulosum* and diallyl disulphide in *A. tuberosum* as described in [31]

Among 50 annotated peaks, 12 metabolite peaks showed significant changes in *A. chinense* and *A. tuberosum* when compared to the control cultivar, *A. fistulosum* (class 01). Dipropyl disulphide (ID101) known as the main VOC component in *Allium* plants was included in the 12 metabolite peaks. The 12 compounds as well as previously reported compounds were listed in Table 4 including thiosulfinates produced from *S*-alk(en)yl cysteine sulfoxides which are sulfur-storage compounds. These peaks are like to be used as discriminative compounds in VOC profiles of *A. chinense*, *A. tuberosum*, and *A. fistulosum*.

**Table 4:** List of previously reported compounds in *Allium* species and 12 VOCs with significant changes in *A. chinense* and *A. tuberosum* against *A. fistulosum* in this study

Name	ID	Detected *Allim* subspecies	Reference
Sulfur-containing peaks			
Thiophene, 2,4-dimethyl	ID032	*A. chinense, A. tuberosum* and *A. fistulosum*	
Dimethyl trisulfide	ID054	*A. chinense, A. tuberosum* and *A. fistulosum*	[42]
3-Thiophenecarboxaldehyde	ID065	*A. chinense, A. tuberosum* and *A. fistulosum*	
Diallyl disulphide	ID091	*A. chinense, A. tuberosum* and *A. fistulosum*	
1,2,4-Trithiolane	ID099	*A. chinense, A. tuberosum* and *A. fistulosum*	
Dipropyl disulfide	ID101	*A. chinense, A. tuberosum* and *A. fistulosum*	
3-Vinyl-1,2-dithiacyclohex-5-ene	ID134	*A. chinense, A. tuberosum* and *A. fistulosum*	
2,5-Thiophenedicarboxaldehyde	ID154	*A. chinense, A. tuberosum* and *A. fistulosum*	
Thieno[2,3-b]thiophene, 2-methyl	ID200	*A. chinense, A. tuberosum* and *A. fistulosum*	
Fully saturated thiosulfinates	ND	*A. cepa, A. sativum, A. ursinum*, *A. porrum, A. fistulosum, A. ascalonicum, A. ampeloprasum*, *A. schoenoprasum* and *A. tuberosum*	[10]
Mono-*S*-b,c-unsaturated thiosulfinates	ND	*A. cepa, A. sativum, A. ursinum*, *A. porrum, A. fi A. m, A, A. ascalonicum, A. ampeloprasum*, *A. schoenoprasum* and *A. tuberosum*	[10]
Di-*S*-b, c-unsaturated thiosulfinates	ND	*A. cepa, A. sativum, A. ursinum*, *A. porrum, A. fistulosum, A. ascalonicum, A. ampeloprasum*, *A. schoenoprasum* and *A. tuberosum*	[10]
Mono-a, b-unsaturated thiosulfinates	ND	*A. cepa, A. sativum, A. ursinum*, *A. porrum, A. fistulosum, A. ascalonicum, A. ampeloprasum*, *A. schoenoprasum* and *A. tuberosum*	[10]
Mixed a, b- and c-unsaturated thiosulfinates.	ND	*A. cepa, A. sativum, A. ursinum*, *A. porrum, A. fistulosum, A. ascalonicum, A. ampeloprasum*, *A. schoenoprasum* and *A. tuberosum*	[10]
Allicin (diallylthiosulphinate)	ND	*A. sativum*	[5]
Alliin (*S*-allyl-l-cysteine sulphoxide)	ND	*A. sativum,A. ursinum, A. ampeloprasum* and *A. longicuspis*	[5, 10]
Dipropyl disulphide	ID101	*A. fistulosum* and *A. tuberosum*	[6, 42]
Dipropyl trisulphide	ND	*A. fistulosum* and *A. tuberosum*	[6]
1-Propenyl propyl disulphide	ND	*A. fistulosum* and *A. tuberosum*	[6]
Methiin (*S*-methyl-*l*-cysteine sulfoxide)	ND	*A.* *cepa, A. sativum,A. chinens* and *A. longicuspis*	[10]
Propiin (*S*-propyl-*l*-cysteine sulfoxide)	ND	*A. cepa, A. porrum, A. porrum, A. altaicum* and *A. fistulosum*	[10]
Isoalliin (*S*-propenyl-*l*-cysteine sulfoxide)	ND	*A. cepa, A. nutans,A. ascalonicum* and *A. schoenoprasum*	[10]
Ethiin (S-ethyl-*l*-cysteine sulfoxide)	ND	*A. aflatunens, A. ampeloprasum, A. ochotense* and *A. victorialis*	[10]
Butiin (S–*n*-butyl-*l*-cysteine sulfoxide)	ND	*A. siculum*	[10]
1-Propenyl-containing disulfides	ND	*A. uictorialis*	[13]
Thiopropanal *S*-oxide	ND	*A. cepa*	[42]
Propenyl propyl disulphide	ND	*A. cepa*	[42]
1-Propenyl propyl disulphide	ND	*A. cepa*	[42]
Di-1-propenyl disulphide	ND	*A. cepa*	[42]
Methyl propyl trisulphide	ND	*A. cepa*	[42]
Propenyl propyl trisulphide	ND	*A. cepa*	[42]
Peaks consisting of CHO			
2-Butenal, 2-ethyl	ID011	*A. chinense, A. tuberosum* and *A. fistulosum*	[13]
Decenal, (2*E*)	ID143	*A. chinense, A. tuberosum* and *A. fistulosum*	[13]
2-Methyl-2-pentenal	ND	*A. uictorialis* and *A. cepa*	[13, 42]
Prop(en)yl aldehydes	ND	*A. cepa*	[42]
2-Methyl-2-pentenal	ND	*A. cepa*	
Peaks consisting of CH			
Nonadecane	ID322	*A. chinense, A. tuberosum* and *A. fistulosum*	[53–55]

*ND* not detected in this study

## Conclusions

Since *Allium* plants emit various types of sulfur-containing compounds and other VOCs, comprehensive profiling techniques are needed. We developed a VOC profiling method for the HS of *Allium* samples that is based on an ITEX technique and our metabolomics pipeline by using GC-TOF–MS [56, 57]. The amount of sample material needed for HS collection was much lower with our ITEX-method than the traditional methods such as solvent extraction and steam distillation. Our findings suggest, that ITEX-based VOC profiling yields good reproducibility for the detection of various types of VOC in *Allium* plants. As ITEX-based VOC profiling captures differences in the composition of VOCs in the HS of *Allium* plants, it is probably appropriate for chemotaxonomic classification of these plants. For odor analysis of samples with strong odors, for example *Allium* plants, GC–olfactometry (GC–O) coupled with MS is useful because it facilitates the evaluation of odor compounds and yields MS spectral information. However, as the concentration of odor compound is often very low and odor-related VOCs can interact synergistically or additively, the identification of actual “odor” peaks remains difficult. Taken together, we think that the HS sampling- and the ITEX-based VOC profiling methods presented here help to improve the detection of odor compounds in *Allium* plants.

## Methods

### Chemicals

All chemicals and reagents used for this study were of spectrometric grade. The *n*-alkane standard solution C8–C20 for determination of RI was purchased from Fluka Chemical (Tokyo, Japan), deuterium-labeled alkanes used to distinguish natural alkanes collected from *Allium* samples were obtained from Cambridge Isotope Laboratories (Andver, USA), and dipropyl disulfide (98 %) and surrogate standard mixture (EPA524.2) from Sigma-Aldrich Japan (Tokyo, Japan). The other chemicals were purchased from Nacalai Tesque (Kyoto, Japan) or Wako Pure Chemical Industries (Osaka, Japan).

### Plant material and sample preparation procedure

Metadata for this study are provided in Additional file 2.

Ten *Allium* (*A*.) *fistulosum* species, six spring onion cultivars, two scallions, and two Japanese-leek cultivars, rakkyo (*A. chinense*) and Oriental garlic (*A. tuberosum*), were purchased from a grocer in Kawasaki, Japan or harvested in a Japanese field (see Table 1 and Additional file 2). After removing the roots, a 10-cm length of the sheath and the basal plate of each plant sample were collected and chopped with stainless steel surgical blades (Feather, Tokyo, Japan). Out of the *A. fistulosum* cultivars, four were grown by applying a method (hilling) similar to that used for growing the leek *A. ampeloprasum* var. *porrum* to obtain longer white stems for consumption in Japan (Fig. 1f, g, h, l). Each sample was immediately frozen in liquid nitrogen and kept at −80 °C until use. As the group of samples of Mikata spring onion (class01) was gathered center of the PCA score scatter plot (Fig. 4), this cultivar was chosen as the control.

The samples were crushed into powder (2 min at 4 °C) in a Mixer Mill MM 311 instrument featuring a grinding jar with a stainless steel screw cap (Restech, Tokyo, Japan) and the frozen powder from each sample (flesh weight, 1 g) was weighed in a 20-ml HS vial (Supelco, MO, USA). For VOC profiling of *Allium* plants we used a modified method of Tikunov et al. [23] and Kusano et al. [31]. Briefly, the 20-ml HS-GC vial (Supelco) containing the frozen powder was closed with a magnetic screw cap (AMR, Tokyo, Japan) for ITEX- and SPME-analysis. Then, 1 ml of 100 mM 2,2′,2′’,2′’’-(ethane-1,2-diyldinitrilo) tetraacetic acid (EDTA)- NaOH water solution (pH 7.5) was added to each vial; the water derived from an *Allium* sample was considered to be equal to 1 ml. After vortexing, 10 μl of solution containing *n*-decane (*d*_*22*_, 99 %; 50 μM), *n*-pentadecane (*d*_*32*_, 98 %; 50 μM), *n*-eicosane (*d*_*42*_, 98 %; 50 μM) for definition of RI and EPA524.2 fortification solution (20 μg/ml of fluorobenzene, 4-bromofluorobenzene, and 1,2-dichlorobenzene-*d*_*4*_) as ISs was mixed in methanol, then solution was added to each vial as IS. Solid CaCl_2_ was added to obtain a final concentration of 5 M and the samples were stored overnight at 22 °C.

### HS collection using the SPME fiber

The SPME device for a CTC CombiPAL auto-sampler (CTC Analytics, Zwingen, Switzerland) was purchased from AMR (Tokyo, Japan). We used an SPME fiber comprised of a 65-μm-thick layer of polydimethylsiloxane (PDMS)/divinylbenzene (DVB)-fused silica (FS) fiber/stainless-steel (SS) tube. Before analysis, the fiber was conditioned at 250 °C for 30 s in the injection port of an Agilent 6890 N gas chromatograph (Agilent Technologies, Wilmington, USA) equipped with a 30 m × 0.25 mm inner diameter fused-silica capillary column with a chemically bound 0.25-μl film Rtx-5 Sil MS stationary phase (RESTEK, Bellefonte, USA). Collection of volatiles was carried out by inserting the SPME-fiber to the vial and by trapping the VOCs for 20 min at 80 °C under continuous agitation. After HS collection it was placed in the injection port of the gas chromatograph that was coupled to a Pegasus III TOF mass spectrometer (LECO, St. Joseph, USA). The thermodesorption of VOCs occurred for 15 s at 250 °C.

### HS collection using the ITEX device

We used a CTC CombiPAL auto-sampler (PAL COMBI-xt) featuring the ITEX device PAL ITEX-2 option (CTC Analytics). The ITEX procedure was controlled with a PAL Cycle Composer (CTC analytics). We conducted preliminary experiments to choose an appropriate sorbent material from the four materials, Tenax TA, Tenax GR (TGR), Carbosieve SIII (CSIII) and mixed TGR and CSIII (TGR/CSIII), that are commercially available (data not shown). Then, we chose that the sorbent material for the ITEX-2 portion was TGR (80/100 mesh)/CSIII (60/80 mesh). The parameters for HS collection were as described in the Additional file 2. After HS collection, 500 μl of the HS sample were injected into the injection port of the gas chromatograph coupled to the mass spectrometer used for HS collection by ITEX.

### GC-TOF–MS analysis

GC-TOF–MS conditions were as described in the Additional file 2. Data acquisition was on a Pegasus III TOF mass spectrometer (LECO); the acquisition rate was 30 spectra/s in the mass range of a mass-to-charge ratio of m/z = 30–550. Five ISs were used for data normalization.

### Data analysis

Raw data were exported in the network common data form (NetCDF) file format using LECO ChromaTOF software (version 2.32) and then processed with the hierarchical multi-curve resolution (H-MCR) method [38]. We obtained the normalized response for calculating the signal intensity of each metabolite from the mass-detector response by using the cross-contribution compensating multiple standard normalization (CCMN) method [44]. The resolved mass spectra were matched against reference mass spectra in the NIST-L (version NIST05) using NIST MS search program (version 2.0, http://www.chemdata.nist.gov/dokuwiki/doku.php?id=chemdata:ms-search). Peaks were tentatively identified according to the guidelines for metabolite identification [58]. When mass spectra exhibited a match value greater than 799 and the corresponding peaks had RIs with small differences upon comparison of their resolved mass spectra and RIs against those in the reference libraries (Ad-L, 3rd and 4th edition, and Te-L) and against Vo and NIST-L (see Table 2 and “Results and discussion” section), the peaks were considered to be putatively annotated compounds. We compared the RIs of sulfur-containing metabolites and compounds we detected with those reported in the literature [51, 52, 59].

To estimate the number of compounds that overlapped with each reference library, we first exported the mass spectral information, including the compound name, RI, synonyms, and *m/z* value, with relative peak intensity (maximum, 999; minimum, one) from each library in ASCII text format (.MSP) automatically. Then we compared the similarity of the mass spectra in each library using MassBank [60]. The similarity (≥850 or 900) and the RI difference (<|30 unit|) were used to extract the same or very similar compounds from the query library and NIST05. It should be noted that the standard deviation (SD) of the absolute RI difference of these compounds is less than 8.8 when we applied similarity of ≥850 (Table 3).

The lower limit of quantification (LLOQ) and the limit of detection (LOD) of dipropyl disulfide obtained from ITEX-GC-TOF–MS- and SPME-GC-TOF–MS analyses were estimated as described in the Additional file 2.

### Statistical analysis

Multivariate analysis was performed with SIMCA-P + 12.0 software (Umetrics AB, Umeå, Sweden). For our analysis, profile data were log_10_-transformed, centered, and scaled to unit variance.

Log_2_-transformed profile data were statistically analyzed using the LIMMA package [61]. It includes FDR correction for multiple testing [62] in the R environment for statistical computing (version 2.14.1 for 64-bit).

## Additional files

10.1186/s13104-016-1942-5 Comparison of the peaks collected with ITEX and SPME devices. a. Total ion chromatograms (TICs) of VOCs in the HS of *A. fistulosum* (class01) obtained by ITEX-GC-TOF–MS (left) and SPME-GC-TOF–MS (right). b. VOC peak annotation with RI information obtained by ITEX-GC-TOF–MS (left) and SPME-GC-TOF–MS (right). c. The PCA score scatter plot of VOC profiles of *A. fistulosum* (class01) obtained by using ITEX-GC-TOF–MS and SPME-GC-TOF–MS. We used three biological replicates (six individual plants per replicate). *AU* arbitrary unit. Fluorobenzene, 4-bromofluorobenzene, and 1,2-dichlorobenzene-*d*_*4*_ were used as ISs. Of these, peaks of luorobenzene and 4-bromofluorobenzene were detected (black arrow in Additional file 1).
10.1186/s13104-016-1942-5 Metabolomic methods.
10.1186/s13104-016-1942-5 Tentatively annotated VOCs in the HS of *Allium* plants collected with the ITEX device. The log_2_-fold change (log_2_FC) was calculated using the LIMMA package (see the “Methods” section). We analyzed three biological replicates of each *Allium* plant except for the white spring onion cultivar (class05, *n* = 2). The FDR was <0.05. *A2* tentatively identified by referring to the libraries (match value >799); *I* identified by comparing the authentic standards.

## References

[1] Dudareva N, Negre F, Nagegowda DA, Orlova I (2006). Plant volatiles: recent advances and future perspectives. Crit Rev Plant Sci.

[2] Knudsen JT, Eriksson R, Gershenzon J, Stahl B (2006). Diversity and distribution of floral scent. Bot Rev.

[3] D’Alessandro M, Turlings TCJ (2006). Advances and challenges in the identification of volatiles that mediate interactions among plants and arthropods. Analyst.

[4] Block E. Garlic and other *Allium*s: The lore and the science: royal society of chemistry. 2009.

[5] Michael K. Volatile compounds of the genus *Allium* L. (Onions). In: Volatile sulfur compounds in food. Am Chem Soc. 2011;1068:183–214.

[6] Slusarenko AJ, Patel A, Portz D (2008). Control of plant diseases by natural products: allicin from garlic as a case study. Eur J Plant Pathol.

[7] Hori M (2007). Onion aphid (*Neotoxoptera formosana*) attractants, in the headspace of *Allium**fistulosum* and *A*. *tuberosum* leaves. J Appl Entomol.

[8] Landry JF (2007). Taxonomic review of the leek moth genus Acrolepiopsis (Lepidoptera: acrolepiidae) in North America. Can Entomol.

[9] Dugravot S, Thibout E (2006). Consequences for a specialist insect and its parasitoid of the response of *Allium* porrum to conspecific herbivore attack. Physiol Entomol.

[10] Thibout E, Guillot JF, Auger J (1993). Microorganisms are involved in the production of volatile kairomones affecting the host-seeking behavior of Diadromus–Pulchellus, a parasitoid of Acrolepiopsis–Assectella. Physiol Entomol.

[11] Rose P, Whiteman M, Moore PK, Zhu YZ (2005). Bioactive *S*-alk(en)yl cysteine sulfoxide metabolites in the genus *Allium*: the chemistry of potential therapeutic agents. Nat Prod Rep.

[12] Scherer C, Jacob C, Dicato M, Diederich M (2009). Potential role of organic sulfur compounds from *Allium* species in cancer prevention and therapy. Phytochem Rev.

[13] Azadi HG, Riazi GH, Ghaffari SM, Ahmadian S, Khaife TJ (2008). Antitumor activity of *Allium* hirtifolium (Iranian shallot) and allicin: microtubule-interaction properties and effects on cancer cell lines. FEBS J.

[14] Nishimura H, Wijaya CH, Mizutani J (1988). Volatile flavor components and antithrombotic agents: vinyldithiins from *Allium* victorialis L. J Agr Food Chem.

[15] Cavallito CJ, Bailey JH (1944). Allicin, the antibacterial principle of *Allium* sativum I Isolation, physical properties, and antibacterial action. J Am Chem Soc.

[16] Kyung KH (2012). Antimicrobial properties of *Allium* species. Curr Opin Biotech.

[17] Bicchi C, Cordero C, Liberto E, Sgorbini B, Rubiolo P (2008). Headspace sampling of the volatile fraction of vegetable matrices. J Chromatogr A.

[18] Palma-Harris C, McFeeters RF, Fleming HP (2001). Solid-phase microextraction (SPME) technique for measurement of generation of fresh cucumber flavor compounds. J Agr Food Chem.

[19] Bicchi C, Cordero C, Liberto E, Rubiolo P, Sgorbini B (2004). Automated headspace solid-phase dynamic extraction to analyse the volatile fraction of food matrices. J Chromatogr A.

[20] Martin A, Farquar G, Jones AD, Frank M (2010). Human breath analysis: methods for sample collection and reduction of localized background effects. Anal Bioanal Chem.

[21] Filipiak W, Sponring A, Baur MM, Filipiak A, Ager C, Wiesenhofer H, Nagl M, Troppmair J, Amann A (2012). Molecular analysis of volatile metabolites released specifically by Staphylococcus aureus and Pseudomonas aeruginosa. BMC Microbiol.

[22] Bicchi C, Ruosi MR, Cagliero C, Cordero C, Liberto E, Rubiolo P, Sgorbini B (2011). Quantitative analysis of volatiles from solid matrices of vegetable origin by high concentration capacity headspace techniques: determination of furan in roasted coffee. J Chromatogr A.

[23] Tikunov Y, Lommen A, de Vos CHR, Verhoeven HA, Bino RJ, Hall RD, Bovy AG (2005). A novel approach for nontargeted data analysis for metabolomics. Large-scale profiling of tomato fruit volatiles. Plant Physiol.

[24] Beaulieu JC, Grimm CC (2001). Identification of volatile compounds in cantaloupe at various developmental stages using solid phase microextraction. J Agr Food Chem.

[25] Oliveira RCS, Oliveira LS, Franca AS, Augusti R (2009). Evaluation of the potential of SPME-GC-MS and chemometrics to detect adulteration of ground roasted coffee with roasted barley. J Food Compos Anal.

[26] Salanţă L, Tofană M, Socaci S, Lazar C, Michiu D, Fărcas A (2012). Determination of the volatile compounds from hop and hop products using ITEX/GC-MS technique. J Agroaliment Process Technol.

[27] Laaks J, Jochmann MA, Schilling B, Schmidt TC (2010). In-tube extraction of volatile organic compounds from aqueous samples: an economical alternative to purge and trap enrichment. Anal Chem.

[28] Jochmann MA, Yuan X, Schilling B, Schmidt TC (2008). In-tube extraction for enrichment of volatile organic hydrocarbons from aqueous samples. J Chromatogr A.

[29] Skogerson K, Wohlgemuth G, Barupal DK, Fiehn O (2011). The volatile compound BinBase mass spectral database. BMC Bioinformatics.

[30] Malowicki SM, Martin R, Qian MC (2008). Volatile composition in raspberry cultivars grown in the Pacific Northwest determined by stir bar sorptive extraction-gas chromatography-mass spectrometry. J Agric Food Chem.

[31] Kusano M, Iizuka Y, Kobayashi M, Fukushima A, Saito K (2013). Development of a direct headspace collection method from arabidopsis seedlings using HS-SPME-GC-TOF-MS analysis. Metabolites.

[32] Laaks J, Jochmann MA, Schilling B, Schmidt TC (2015). Optimization strategies of in-tube extraction (ITEX) methods. Anal Bioanal Chem.

[33] Veriotti T, Sacks R (2000). High speed GC/MS of gasoline-range hydrocarbon compounds using a pressure-tunable column ensemble and time-of-flight detection. Anal Chem.

[34] Stein SE, Ausloos P, Clifton CL, Klassen JK, Lias SG, Mikaya AI, Sparkman OD, Tchekhovskoi DV, Zaikin V, Zhu D (1999). Evaluation of the NIST/EPA/NIH mass spectral library. Abstr Pap Am Chem S.

[35] Adams RP. Identification of essential oil components by gas chromatography/quadrupole mass spectroscopy: Allured Pub. Corporation. 2001.

[36] Stein SE (1999). An integrated method for spectrum extraction and compound identification from gas chromatography/mass spectrometry data. J Am Soc Mass Spectr.

[37] Hoffmann N, Stoye J (2009). ChromA: signal-based retention time alignment for chromatography–mass spectrometry data. Bioinformatics.

[38] Jonsson P, Johansson ES, Wuolikainen A, Lindberg J, Schuppe-Koistinen I, Kusano M, Sjostrom M, Trygg J, Moritz T, Antti H (2006). Predictive metabolite profiling applying hierarchical multivariate curve resolution to GC–MS data—a potential tool for multi-parametric diagnosis. J Proteome Res.

[39] Lommen A (2009). MetAlign: interface-driven, Versatile metabolomics tool for hyphenated full-scan mass spectrometry data preprocessing. Anal Chem.

[40] Luedemann A, Strassburg K, Erban A, Kopka J (2008). TagFinder for the quantitative analysis of gas chromatography–mass spectrometry (GC-MS)-based metabolite profiling experiments. Bioinformatics.

[41] Smith CA, Want EJ, O’Maille G, Abagyan R, Siuzdak G (2006). XCMS: processing mass spectrometry data for metabolite profiling using Nonlinear peak alignment, matching, and identification. Anal Chem.

[42] Jarvenpaa EP, Zhang ZY, Huopalahti R, King JW (1998). Determination of fresh onion (*Allium* cepa L.) volatiles by solid phase microextraction combined with gas chromatography mass spectrometry. Z Lebensm Unters F A.

[43] Gullberg J, Jonsson P, Nordstrom A, Sjostrom M, Moritz T (2004). Design of experiments: an efficient strategy to identify factors influencing extraction and derivatization of Arabidopsis thaliana samples in metabolomic studies with gas chromatography/mass spectrometry. Anal Biochem.

[44] Redestig H, Fukushima A, Stenlund H, Moritz T, Arita M, Saito K, Kusano M (2009). Compensation for systematic cross-contribution improves normalization of mass spectrometry based metabolomics data. Anal Chem.

[45] Kolb B, Ettre LS. Static headspace–gas chromatography: theory and practice: John Wiley and Sons; 2006.

[46] Eady CC, Kamoi T, Kato M, Porter NG, Davis S, Shaw M, Kamoi A, Imai S (2008). Silencing onion lachrymatory factor synthase causes a significant change in the sulfur secondary metabolite profile. Plant Physiol.

[47] Laaks J, Jochmann MA, Schmidt TC (2012). Solvent-free microextraction techniques in gas chromatography. Anal Bioanal Chem.

[48] Zapata J, Mateo-Vivaracho L, Lopez R, Ferreira V (2012). Automated and quantitative headspace in-tube extraction for the accurate determination of highly volatile compounds from wines and beers. J Chromatogr A.

[49] Pino JA, Fuentes V, Correa MT (2001). Volatile constituents of Chinese chive (*Allium**tuberosum* Rottl. ex Sprengel) and rakkyo (*Allium**chinense* G. Don). J Agric Food Chem.

[50] Shen CX, Xiao H, Parkin KL (2002). In vitro stability and chemical reactivity of thiosulfinates. J Agr Food Chem.

[51] Block E, Putman D, Zhao SH (1992). *Allium* chemistry–Gc Ms analysis of thiosulfinates and related-compounds from onion, leek, scallion, shallot, chive, and Chinese chive. J Agr Food Chem.

[52] Kuo MC, Ho CT (1992). Volatile constituents of the distilled oils of Welsh onions (*Allium**fistulosum* L. variety maichuon) and scallions (*Allium**fistulosum* L. variety caespitosum). J Agr Food Chem.

[53] Yusuf OK, Bewaji CO (2011). Evaluation of essential oils composition of methanolic *Allium**sativum* extract on Trypanosoma brucei infected rats. Res Pharm Biotech.

[54] Kays SJ, Hatch J, Yang DS (2005). Volatile floral chemistry of Heliotropium arborescens L. ‘Marine’. HortScience.

[55] Flamini G, Cioni PL, Morelli I (2003). Use of solid-phase micro-extraction as a sampling technique in the determination of volatiles emitted by flowers, isolated flower parts and pollen. J Chromatogr A.

[56] Kusano M, Fukushima A, Kobayashi M, Hayashi N, Jonsson P, Moritz T, Ebana K, Saito K (2007). Application of a metabolomic method combining one-dimensional and two-dimensional gas chromatography-time-of-flight/mass spectrometry to metabolic phenotyping of natural variants in rice. J Chromatogr B Analyt Technol Biomed Life Sci.

[57] Kusano M, Fukushima A, Arita M, Jonsson P, Moritz T, Kobayashi M, Hayashi N, Tohge T, Saito K (2007). Unbiased characterization of genotype-dependent metabolic regulations by metabolomic approach in Arabidopsis thaliana. BMC Syst Biol.

[58] Sumner LW, Amberg A, Barrett D, Beale MH, Beger R, Daykin CA, Fan TWM, Fiehn O, Goodacre R, Griffin JL (2007). Proposed minimum reporting standards for chemical analysis. Metab Off J Metab Soc.

[59] Kuo MC, Ho CT (1992). Volatile constituents of the solvent extracts of Welsh onions (*Allium**fistulosum* L. variety maichuon) and scallions (A. *fistulosum* L. variety caespitosum). J Agr Food Chem.

[60] Horai H, Arita M, Kanaya S, Nihei Y, Ikeda T, Suwa K, Ojima Y, Tanaka K, Tanaka S, Aoshima K (2010). MassBank: a public repository for sharing mass spectral data for life sciences. JMS.

[61] Smyth GK. Linear models and empirical bayes methods for assessing differential expression in microarray experiments. Statistical applications in genetics and molecular biology. 2004;3:Article3. 10.2202/1544-6115.1027 16646809

[62] Benjamini Y, Hochberg Y (1995). Controlling the false discovery rate: a practical and powerful approach to multiple testing. J Roy Stat Soc.

