# Supplementary figures and images for: Unbiased profiling of volatile organic compounds in the headspace of Allium plants using an in-tube extraction device
Source: BMC Res Notes. 2016 Feb 29;9:133. doi: 10.1186/s13104-016-1942-5 (PMC4772445; doi:10.1186/s13104-016-1942-5)

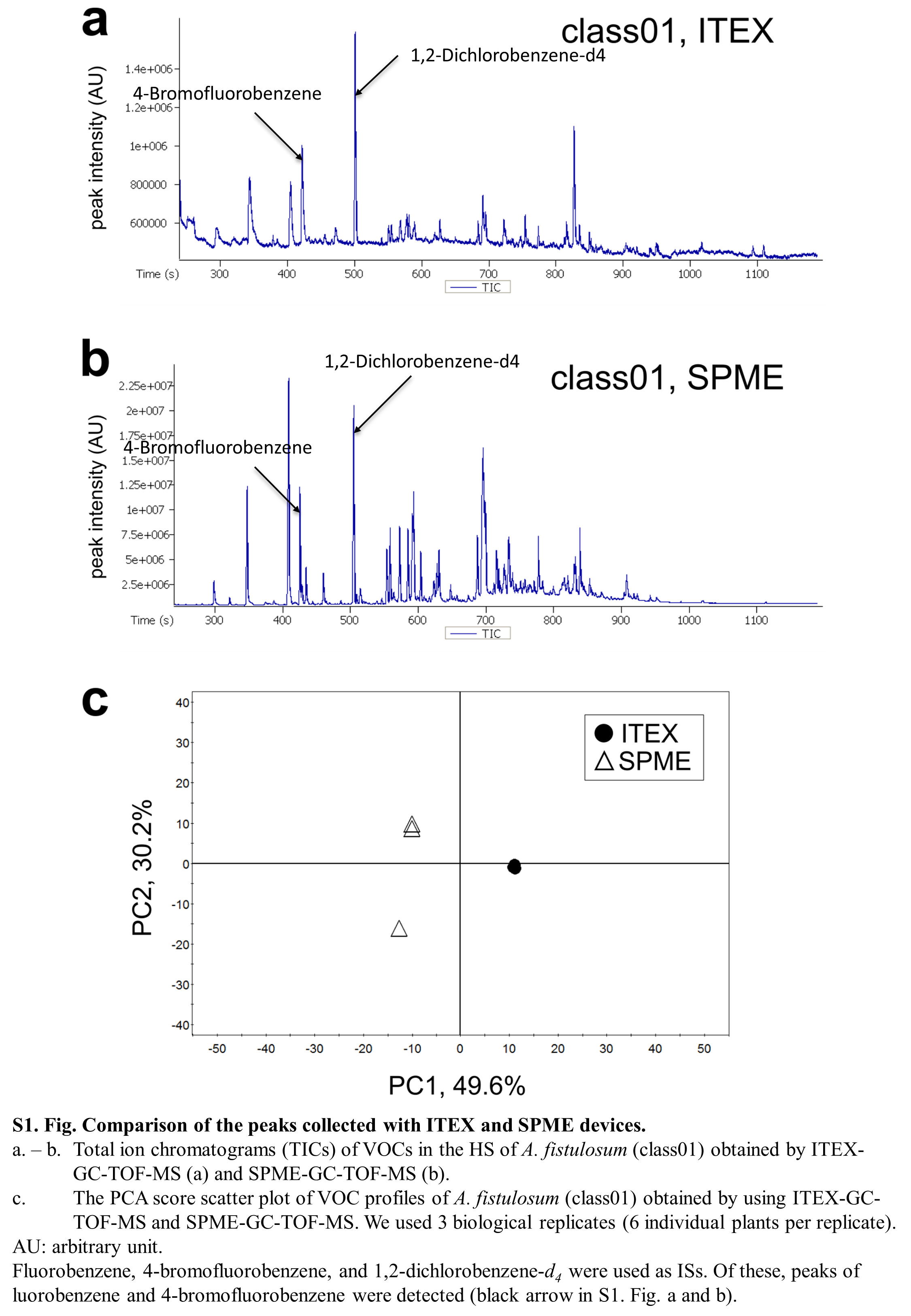

Supplement: Supplementary file 1 — 10.1186/s13104-016-1942-5 Comparison of the peaks collected with ITEX and SPME devices. a. Total ion chromatograms (TICs) of VOCs in the HS of A. fistulosum (class01) obtained by ITEX-GC-TOF–MS (left) and SPME-GC-TOF–MS (right). b. VOC peak annotation with RI information obtained by ITEX-GC-TOF–MS (left) and SPME-GC-TOF–MS (right). c. The PCA score scatter plot of VOC profiles of A. fistulosum (class01) obtained by using ITEX-GC-TOF–MS and SPME-GC-TOF–MS. We used three biological replicates (six individual plants per replicate). AU arbitrary unit. Fluorobenzene, 4-bromofluorobenzene, and 1,2-dichlorobenzene-d 4 were used as ISs. Of these, peaks of luorobenzene and 4-bromofluorobenzene were detected (black arrow in Additional file 1). [file 13104_2016_1942_MOESM1_ESM.tiff]
