# Supplementary material for: Unbiased profiling of volatile organic compounds in the headspace of Allium plants using an in-tube extraction device
Source: BMC Res Notes. 2016 Feb 29;9:133. doi: 10.1186/s13104-016-1942-5 (PMC4772445; doi:10.1186/s13104-016-1942-5)
Supplement: Supplementary file 2 — 10.1186/s13104-016-1942-5 Metabolomic methods. [file 13104_2016_1942_MOESM2_ESM.doc]

# **S1 Methods**

## Metabolomic methods

Based on the recommendation of the Metabolomics Standards Initiative , metadata in this study was shown as follows.

## Plant context metadata

*BioSource Species*:

Ten *Allium fistulosum* species consisting of 6 spring onion cultivars, two scallions, and two Japanese-leek cultivars, rakkyo (Allium chinense), and Oriental garlic (*Allium tuberosum*). Details were summarized in Table 1*.*

*Organ*:

Leaf sheath and basal plate

*Organ specification*:

The 10-cm length of the sheath and basal plate after removal of the roots

*Amount*: Frozen powder (1 g flesh weight)

*Growth condition*:

The samples were purchased from a local grocery store (LAZONA Kawasaki plaza) in Kawasaki, Japan or harvested at the field in Nagahama, Japan

*Experimental condition*:

A part of the samples of *A. fistulosum* (class01) were stored for 15 days at 10 ^o^C under darkness. In this case, we pooled 6 biological replicates as one batch. We analyzed three batches of the cultivar.

*Sampling and sampling date*:

The *Allium* plants were obtained on 15th or 16th October, 2012. The 10 cm length of sheath and basal plate of each sample was chopped by using stainless steel surgical blades (Feather, Tokyo, Japan). We prepared 3 biological replicates of each *Allium* plant except for the cultivar, which was a white spring onion (class05, *n* = 2).

*Metabolism quenching method*:

All samples were frozen within 30 sec in liquid nitrogen after chopping. The samples were kept at -80°C until use.

## Chemical analysis metadata

## Sample preparation

*Sample processing*:

Samples were crushed to powder using grinding jars for a Mixer Mill MM 311 (Restech, Tokyo, Japan) with screw cap made by stainless steel (capacity 15 ml or 50 ml; Restech) at a frequency of 15 Hz for 2 min (cf. the jars were switched every 1 min) at 4°C.

The frozen powder of each sample (1 g flesh weight) was weighed in a 20-ml HS vial (Supelco, Missouri, USA). We followed the procedure of VOC profiling of *Allium* plants as described by and with modifications. The frozen powder in a 20-ml HS-GC vial (Supelco, Missouri, USA) was closed with a magnetic screw cap (18 mm, PTFE/red chlorobutyl; AMR, Tokyo, Japan) for in-tube extraction (ITEX) or a magnetic screw cap (18 mm, Silicon/PTFE; AMR) for solid phase microextraction (SPME). Then, 1 ml of 100 mM 2,2’,2’’,2’’’-(ethane-1,2-diyldinitrilo)tetraacetic acid–sodium hydroxide (EDTA-NaOH) water solution (pH 7.5) was added in each vial. The vial was vortexed to dissolve ice of frozen powder. In this case, the water derived from an *Allium* sample was considered to be equal to 1 ml. After vortexing, 10 μl of internal standard solution containing *n*-decane (*d_22_*, 99%; 50 μM), *n*-pentadecane (*d_32_*, 98%; 50 μM), *n*-eicosane (*d_42_*, 98%; 50 μM) and EPA524.2 fortification solution (20μg/ml of fluorobenzene, 4-bromofluorobenzene and 1,2-dichlorobenzene-*d_4_*) in methanol was added to the vials as internal standards (ISs). Solid CaCl_2_ was added to vials to a final concentration of 5 M. The vial was closed and then sonicated at a frequency of 38 kHz for 10 min using a sonicator (US-108; NSD, Suwa, Japan). Samples were stored overnight at 22°C. The next day we analyzed these samples.

To estimate the lower limit of quantification (LLOQ) and the limit of detection (LOD) of dipropyl disulfide in headspace of HS vials using ITEX-GC-TOF-MS and SPME-GC-TOF-MS, we added 10 μl of dipropyl disulfide (0.25 μM, 2.5μM, 25μM and 250 μM, respectively) to each 20-ml HS vial containing 2 ml of milli-Q water and the ISs. We prepared 3 independent replicates for each concentration.

*Headspace (HS) collection using the SPME fiber*:

A SPME device for a CTC CombiPAL auto-sampler (CTC analytics, Zwingen, Switzerland) was purchased from AMR (Tokyo, Japan). We used an SPME fiber, which was a 65-μm-thick layer of polydimethylsiloxane (PDMS)/divinylbenzene (DVB) -fused silica (FS) fiber/stainless steel (SS) tube for the study. The fiber was conditioned at 250°C for 0.5 h in the injection port of an Agilent 6890N gas chromatograph (Agilent Technologies, Wilmingston, USA) equipped with a 30 m × 0.25 mm inner diameter fused-silica capillary column with a chemically bound 0.25-μl film Rxi-5 Sil MS stationary phase (RESTEK, Bellefonte, USA) before analysis. The fiber was exposed to the vial headspace for 20 min at 80°C under continuous agitation. After HS collection, the fiber was placed into the injection port of the gas chromatograph that was hyphenated with a Pegasus III TOF mass spectrometer (LECO, St. Joseph, USA). The volatile compounds were thermally desorbed for 0.25 min at 250°C for the fiber. After every injection, the fiber was bake out by Needle heater (CTC analytics) for 15 min at 250°C to clean and ready for the next sample.

*HS collection using the ITEX device:*

A CTC CombiPAL autosampler (PAL COMBI-xt) equipped with the ITEX device PAL ITEX-2 option was used (CTC analytics). The ITEX extraction procedure was controlled by using PAL Cycle Composer (CTC analytics). We used Tenax GR (80/100 mesh)/Carbosieve S III (60/80 mesh) (TENAXGR/CSIII) as a sorbent material for the ITEX-2 potion. The parameters for HS collection were as follows: incubation temperature, 80°C; incubation time, 15 min; agitator speed, 500 rpm; number of extraction strokes, 60; syringe temperature, 45°C and 85°C; extraction volume, 1000 μl; extraction speed, 150 μl/s; desorption temperature, 260°C; desorption speed, 100 μl/s; trap cleaning temperature, 260°C; and trap cleaning time, 10 min. After HS collection, 500 μl of the HS sample was injected into the injection port of the gas chromatograph hyphenated with the mass spectrometer that was used for HS collection by SPME.

## GC-TOF-MS conditions

HS sample was injected by an CTC CombiPAL autosampler (CTC analytics) into the injection port of the gas chromatograph (Agilent Technologies) equipped with a 30 m × 0.25 mm inner diameter fused-silica capillary column with ah chemically bound 0.25-μl ﬁlm Rxi-5 Sil MS stationary phase (RESTEK, Bellefonte, USA). A mass spectrometer column change interface (ms NoVent-J; SGE, Yokohama, Japan) was used to prevent air and water from entering the MS during column change-over. Helium was used as the carrier gas at a constant ﬂow rate of 1 ml/min. Septum purge flow and time was set at 20 ml/min and 30 sec. Total flow was set at 20.9 ml/min. Gas saver was used and the flow was set at 15 ml/min. The inlet temperature was 230°C. The temperature program for the HS-VOC analysis started with a 2-min isothermal step at 45°C and this was followed by temperature ramping at 15°C to a final temperature of 250°C, which was maintained for 3.5 min. The transfer line and the ion source temperatures were 250°C and 200°C, respectively. Ions were generated by a 70-eV electron beam. Detector voltage was 1740-eV. The acceleration voltage was turned on after a solvent delay of 180 s.

Data acquisition was performed on a Pegasus III TOF mass spectrometer (LECO) at an acquisition rate of 30 spectra/s in the mass range of a mass-to-charge ratio of m/z = 30–550. Five internal standards were used for data normalization (see Results and Discussion section). Vials containing only the IS mixture were analyzed every 6 samples. Five-μl of alkane standard mixtures (C8–C20 and C21–C40, respectively) were put into the 20-ml of HS-GC vial. The IS mixture as well as the alkane standard mixture were used for calculating the retention indices (RIs) . Vials containing only the IS mixture were analyzed in every six samples for quality control.

## Data processing

*Data processing for GC-MS data*:

The dipropyl disulfide peak in each analyte was processed using Leco ChromaTOF software ver. 3.22 to estimate LLOQ and LOD. We employed definition of LLOQ and LOD described by Sumner et al .

For HS-VOC profiling, non-processed MS data from GC-TOF-MS analysis were exported in the NetCDF format generated by chromatography processing and mass spectral deconvolution software, Leco ChromaTOF version 2.32 (LECO, St. Joseph, USA) to MATLAB 7.0 (Mathworks, Natick, USA), where all data-pretreatment procedures, such as smoothing, alignment, time-window setting, and the hierarchical multi-curve resolution (H-MCR), were carried out . We used the specific m/z area for overlapped peaks instead of the corresponding extracted peaks by the H-MCR method . To normalize peaks, we calculated area of the selected mass spectral values for each internal standards using MATLAB R2011b (Mathworks) with the custom script. Then, the peaks in the data matrix was normalized by the CCMN method .

The resolved MS spectra obtained from the H-MCR method were matched against reference mass spectra using the NIST mass spectral search program for the NIST/EPA/NIH mass spectral library (version 2.0) and our custom software for peak annotation written in JAVA. Peaks were tentatively identified according to the guideline of metabolite identification . When mass spectra showed a match value greater than 799 and the corresponding peaks had RIs with small differences (< |30 unit|) by comparing their resolved mass spectra and RIs to those in the reference libraries, i.e., Adams library (the 3rd and the 4th edition), Terpenoids library (http://massfinder.com/wiki/Terpenoids_Library), VocBinBase , and NIST05 (see Results and Discussion section), the peaks were considered to be putatively annotated compounds. Of these, identification was performed by comparing RIs and mass spectra with those of authentic standards. We compared the RIs of sulfur-containing metabolites and compounds detected in the study with those in the literature .

## Statistical analysis

SIMCA-P+ 12.0 software (Umetrics AB, Umeå, Sweden) was used for multivariate analysis. The profile data were log_10_-transformed, centered, and scaled to unit variance for the analysis.

The profile data were log_2_-transformed, and then statistically analyzed using the LIMMA package , which includes false discovery rate (FDR) correction for multiple testing in the R environment for statistical computing (version 2.14.1 for 64-bit).

## References

1. Bais P, Moon SM, He K, Leitao R, Dreher K, Walk T, et al. PlantMetabolomics.org: a web portal for plant metabolomics experiments. Plant physiology. 152(4):1807-16.

2. Tikunov Y, Lommen A, de Vos CHR, Verhoeven HA, Bino RJ, Hall RD, et al. A novel approach for nontargeted data analysis for metabolomics. Large-scale profiling of tomato fruit volatiles. Plant Physiol. 2005;139(3):1125-37.

3. Kusano M, Iizuka Y, Kobayashi M, Fukushima A, Saito K. Development of a direct headspace collection method from Arabidopsis seedlings using HS-SPME-GC-TOF-MS analysis. Metabolites. 2013;3(2):223-42.

4. Wagner C, Sefkow M, Kopka J. Construction and application of a mass spectral and retention time index database generated from plant GC/EI-TOF-MS metabolite profiles. Phytochemistry. 2003;62(6):887-900.

5. Schauer N, Steinhauser D, Strelkov S, Schomburg D, Allison G, Moritz T, et al. GC-MS libraries for the rapid identification of metabolites in complex biological samples. FEBS Lett. 2005;579(6):1332-7.

6. Sumner LW, Amberg A, Barrett D, Beale MH, Beger R, Daykin CA, et al. Proposed minimum reporting standards for chemical analysis. Metabolomics : 2007;3(3):211-21.

7. Jonsson P, Johansson AI, Gullberg J, Trygg J, A J, Grung B, et al. High-throughput data analysis for detecting and identifying differences between samples in GC/MS-based metabolomic analyses. Anal Chem. 2005;77(17):5635-42.

8. Jonsson P, Johansson ES, Wuolikainen A, Lindberg J, Schuppe-Koistinen I, Kusano M, et al. Predictive metabolite profiling applying hierarchical multivariate curve resolution to GC-MS data--a potential tool for multi-parametric diagnosis. J Proteome Res. 2006;5(6):1407-14.

9. Redestig H, Fukushima A, Stenlund H, Moritz T, Arita M, Saito K, et al. Compensation for systematic cross-contribution improves normalization of mass spectrometry based metabolomics data. Anal Chem. 2009;81(19):7974-80.

10. Adams RP. Identification of essential oil components by gas chromatography/quadrupole mass spectroscopy: Allured Pub. Corporation; 2001.

11. Skogerson K, Wohlgemuth G, Barupal DK, Fiehn O. The volatile compound BinBase mass spectral database. BMC bioinformatics. 2011;12:321.

12. Stein SE, Ausloos P, Clifton CL, Klassen JK, Lias SG, Mikaya AI, et al. Evaluation of the NIST/EPA/NIH Mass Spectral Library. Abstr Pap Am Chem S. 1999;218:U368-U. P.

13. Kuo MC, Ho CT. Volatile constituents of the solvent extracts of Welsh onions (Allium fistulosum L. variety maichuon) and scallions (A. fistulosum L. variety caespitosum). J Agr Food Chem. 1992;40(10):1906-10.

14. Kuo MC, Ho CT. Volatile constituents of the distilled oils of Welsh onions (Allium fistulosum L. variety maichuon) and scallions (Allium fistulosum L. variety caespitosum). J Agr Food Chem. 1992;40(1):111-7.

15. Block E, Putman D, Zhao SH. Allium chemistry - GC MS analysis of thiosulfinates and related compounds from onion, leek, scallion, shallot, chive, and Chinese chive. J Agr Food Chem. 1992;40(12):2431-8.

16. Smyth GK. Linear models and empirical Bayes methods for assessing differential expression in microarray experiments. Statistical applications in genetics and molecular biology. 2004;3:Article3.

17. Benjamini Y, Hochberg Y. Controlling the false discovery rate: A practical and powerful approach to multiple testing. Journal of the Royal Statistical Society. 1995;B57:289-300.
